# Valve-Sparing Aortic Root Replacement: Comparison of Long-Term Outcomes Between the David and Yacoub Procedure in Denmark

**DOI:** 10.1093/icvts/ivag107

**Published:** 2026-04-10

**Authors:** Emil Johannes Ravn, Lytfi Krasniqi, Poul Erik Mortensen, Bo Juel Kjeldsen, Jens Lund, Oke Gerke, Morten Holdgaard Smerup, Ivy Susanne Modrau, Jordi Sanchez Dahl, Lars Peter Schødt Riber

**Affiliations:** Department of Cardiothoracic Surgery, Odense University Hospital, Odense 5000, Denmark; Department of Cardiology, Odense University Hospital, Odense 5000, Denmark; Department of Cardiothoracic Surgery, Odense University Hospital, Odense 5000, Denmark; Department of Clinical Research, University of Southern Denmark, Odense 5000, Denmark; Department of Cardiothoracic Surgery, Odense University Hospital, Odense 5000, Denmark; Department of Cardiothoracic Surgery, Odense University Hospital, Odense 5000, Denmark; Department of Cardiothoracic Surgery, Odense University Hospital, Odense 5000, Denmark; Department of Clinical Research, University of Southern Denmark, Odense 5000, Denmark; Department of Nuclear Medicine, Odense University Hospital, Odense 5000, Denmark; Department of Cardiothoracic Surgery, Copenhagen University Hospital—Rigshospitalet, Copenhagen 2100, Denmark; Department of Cardiothoracic Surgery, Aarhus University Hospital, Aarhus 8200, Denmark; Department of Clinical Medicine, Aarhus University, Aarhus 8200, Denmark; Department of Cardiology, Odense University Hospital, Odense 5000, Denmark; Department of Cardiovascular Medicine, Mayo Clinic, Rochester, MN 55905, United States; Department of Cardiothoracic Surgery, Odense University Hospital, Odense 5000, Denmark; Department of Clinical Research, University of Southern Denmark, Odense 5000, Denmark

**Keywords:** valve-sparing aortic root replacement, aortic root replacement, composite root replacement, David procedure, Yacoub procedure, long-term outcomes

## Abstract

**Objectives:**

The study aimed to compare long-term outcomes of the David and Yacoub valve-sparing aortic root replacement (VSARR) procedures in a nationwide, low-volume setting.

**Methods:**

All patients undergoing elective VSARR in Denmark between January 2010 and April 2022 were identified using the Danish registries. The primary end-point was all-cause death. Secondary end-points included stroke, reoperation, a composite of death or reoperation, and long-term echocardiographic outcomes. Analyses were performed in both crude and propensity score-matched (PSM) populations and compared with outcomes from a control group comprised of all elective composite root replacement procedures performed at the contributing centres in the study period.

**Results:**

A total of 160 patients underwent VSARR (median age; David vs Yacoub; 49.7 [SD 14.1] years vs 51.5 [SD 14.3] years; *P* = .42) with the David (*n* = 92, 57.5%; median follow-up 8.2 [SD 3.6] years) or Yacoub (*n* = 68, 42.5%; median follow-up 7.1 [SD 2.4] years) procedure. Ten-year all-cause death was similar (David 9.3% [95% CI: 2.3-15.2] vs Yacoub 8.8% [95% CI: 3.6-20.6]; log-rank *P* = .54). Reoperation rates (log-rank *P* = .12), stroke incidence (*P* = .41), and the composite end-point of death or reoperation (log-rank *P* = .63) were comparable, with consistent findings in the PSM cohort. Long-term echocardiographic follow-up demonstrated no difference in recurrent regurgitation, stenosis, or valve-related complications. Compared to elective composite root replacement (CRR) (*n* = 486), VSARR demonstrated lower rate of all-cause death (*P* = .027) and stroke (*P* = .026), while reoperation rate was higher (13.8% vs 4.3%; *P* < .001). However, the risk of reoperation was comparable between procedures when accounting for competing risk of death (log-rank *P* = .17).

**Conclusions:**

Long-term outcomes following elective VSARR using the David and Yacoub procedure were comparable. VSARR was associated with lower risk of all-cause death and stroke compared to elective CRR, supporting the two VSARR procedures as durable options when performed in experienced centres with careful patient selection, even in low-volume settings.

## Graphical Abstract

**Figure:**
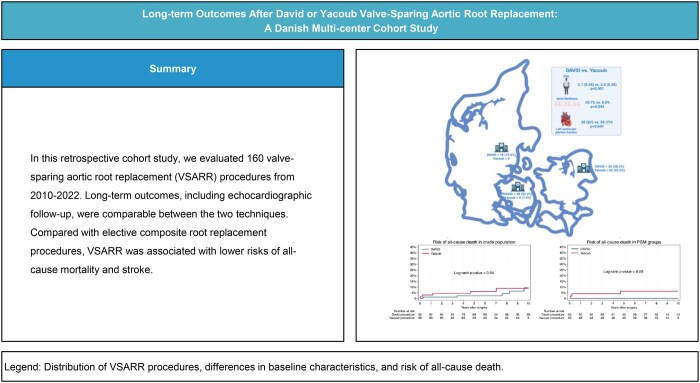

## INTRODUCTION

Valve-sparing aortic root replacement (VSARR) is a highly specialized surgical treatment of aortic root aneurysms, which preserve the native aortic valve to avoid anticoagulation therapy. The most common and widely used VSARR techniques are remodelling and reimplantation of the native aortic valve.^1^^,^^2^ The Yacoub procedure remodels the aortic root with a synthetic graft, creating neosinuses while preserving preoperative annular dimensions.^2^ However, the preservation of the aortic annulus have raised concerns about increased risk of postoperative annular dilatation and recurrent aortic regurgitation (AR), which is more properly addressed in the David procedure. The David procedure reimplants the aortic valve within a synthetic graft and thus supports the annulus and sinotubular junction.^3^ However, this technique encloses the interleaflet triangles, which may limit root expansibility and valve motion, potentially causing more stress on the native aortic valve.^1^ Modified versions of the techniques have since been developed to address their individual disadvantages,^4^^,^^5^ yielding satisfactory short- and mid-term results.^6^^,^^7^

Despite different surgical techniques, the European Society of Cardiology (ESC) and the European Association for Cardio-Thoracic Surgery (EACTS) guidelines do not favour one technique over the other, but generally recommend VSARR for younger patients when performed at experienced centres.^8^ There is a paucity in evidence evaluating differences between VSARR techniques based on nationwide data and low-volume settings, while the current literature is often based on limited follow-up.^6–8^ VSARR was implemented in Denmark two decades ago, though institutional volumes still remain relatively low,^9^ but with satisfying results.^10^ Thus, the aim of this nationwide registry-based, observational cohort study was to compare long-term risk of all-cause death, reoperation, stroke, and valve function following VSARR with the David or Yacoub procedure in Denmark. By including all tertiary centres and validating registry data against operative and clinical records, this study provides robust real-world evidence on long-term outcomes of VSARR in a low-volume setting.

## METHODS

### Ethical consideration

The study was approved by the Region of Southern Denmark’s record of data processing activities (Journal nr.: 22/26135; 22/26364), Odense, Denmark, on June 13, 2022. According to Danish legislation for registry-based research using anonymized data, individual informed consent is not required and was therefore waived. No biological material or biobank data were used in this study. The study followed the Reporting of Observational studies in Epidemiology reporting (STROBE) guidelines.^11^

### Setting, participants, and exposures

We included all patients undergoing VSARR with either David or Yacoub procedure in Denmark from January 2010 to April 2022. No patients were excluded from our analyses. Patients were categorized according to VSARR procedure and were followed until death, migration, or the end of the follow-up period on November 24, 2025, whichever occurred first. The two groups were also compared in propensity score-matched (PSM) cohorts.

### Outcomes and missing data

The primary outcome was risk of all-cause death. Secondary outcomes included the rates of stroke, risk of reoperation, and the composite end-point of risk of all-cause death or reoperation, along with echocardiographic measures such as left ventricular ejection fraction (LVEF), recurrent AR, and aortic stenosis (AS) at discharge, 3-5 years and beyond 5 years following surgical treatment with VSARR.

All definitions, end-points, International Classification of Diseases (ICD) 10 codes, and procedure codes are reported in **Table S1** in the **Appendix**. Missing data are clearly indicated in the tables using brackets“”.

### Data source

The Danish registries used in this study are well-known for their high quality, with their methodologies and accuracy thoroughly documented in the literature.^12–16^ The study population was identified using the prospective Western Danish Heart Registry (WDHR),^13^^,^^17^ and the prospective Danish Heart Registry (DHR).^18^ The WDHR contains data from three of the four national tertiary cardiac centres (Odense University Hospital, Aarhus University Hospital, and Aalborg University Hospital), while the DHR contains data from all four tertiary centres, including Rigshospitalet. The two databases are continuously maintained and encompass comprehensive information regarding pre-existing conditions as well as perioperative and postoperative data on all patients undergoing cardiac procedures at the four centres.^13^^,^^18^ The validation process has been previously published.^9^ Patients from Rigshospitalet were identified using the DHR and merged with patients identified in the WDHR. All data were validated and adjudicated according to the standards of the WDHR.

Death dates were obtained from the Civil Registration System.^14^ The Danish Register of Causes of Death supplied data on death, including dates and both primary and secondary causes of death, as certified by physicians.^16^

Follow-up data on postoperative outcomes were retrieved from the validated and highly reliable National Danish Patient Registry.^12^ The Danish National Patient Registry records all hospital interactions for Danish citizens since 1978, requiring a primary discharge diagnosis at discharge in accordance with the ICD 10.^12^ Additionally, the Danish National Patient Registry has included data on surgical procedures since 1996.^15^ Cases registered with stroke or reoperation were further adjudicated by review of medical records and reported according to VARC-3.^19^

Echocardiographic measures for the crude population, including LVEF, recurrent AR, AS, aortic mean gradient, and valve-related complications, were assessed through a systematic review of echocardiographic reports documented in patients’ medical records. To ensure consistency, data were extracted from standardized reports when available, while acknowledging variability in reporting and potential missing data as limitations.

### Statistics

Continuous data were reported as mean ± SD if normally distributed, and as median (interquartile range, IQR) depending on the shape of the distribution, as assessed by histograms including approximating normal distributions. Categorical data were reported as proportions. Inter-group comparisons were performed using Student’s *t* test or Wilcoxon rank sum test, Chi-squared test, or Fisher’s exact test, as appropriate.

Frailty assessment utilized the Charlson Comorbidity Index,^20^ calculated with Quan Coding Algorithms from the 1987 model for ICD-10 comorbidities (**Table S2**).^21^

Propensity score matching was used to balance baseline characteristics between the two groups, in which the treatment variable (David vs Yacoub) was the dependent variable with EuroSCORE II as the matching outcome. The score was generated with five clinically relevant baseline characteristics and a calliper of 0.2.^22^ Matching method is described in detail in the supplement (**Table S3**).

Time-to-events were calculated as the time from the date of VSARR to the date of the outcome in question. Time-to-event analyses was performed using non-parametric Kaplan-Meier plots as well as log-rank tests and semi-parametrical Cox proportional hazards regression. Schoenfeld residuals were used to assess proportional hazard assumptions, and any violations were reported. Point estimates are supplemented by respective 95% CIs, when appropriate. Analyses were performed for both the crude and PSM groups, including a sensitivity analysis comparing all David procedures to those with the modified Yacoub procedure.

A multivariable model was developed to evaluate the association between VSARR and all-cause death. Covariates were selected *a priori* based on established clinical relevance and included age, sex, body mass index, body surface area, EuroSCORE II, Charlson Comorbidity Index, hypertension, diabetes, dyslipidaemia, creatinine clearance, COPD, and connective tissue disease. Complete case analyses were performed.

*P*-value of less than .05 are considered statistically significant and thus reported with 3 decimals. Statistical analysis is performed with STATA/IC 18 (StataCorp, College Station, Texas 77845, United States).

## RESULTS

We identified 160 elective patients in the WDHR and the DHR, who underwent VSARR with either the David procedure (*n* = 92, 57.5%) or Yacoub procedure (*n* = 68, 42.5%) between January 2010 and April 2022. All procedures were performed in three out of four tertiary centres. The number of VSARR procedures according to centre were: Rigshospitalet (David = 26 [28.3%], Yacoub = 63 [92.6%]), Odense University hospital (David = 48 [52.2%], Yacoub = 5 [7.4%]), and Aarhus University hospital (David = 18 [19.6%], Yacoub = 0) (Graphical Abstract).

Overall, there was no difference in age, sex, body mass index, connective tissue disease, previous cardiac surgery, symptom burden along with comorbidity burden, and operative risk measured by Charlston Comorbidity Index and EuroSCORE II, respectively. Patients undergoing VSARR with the David procedure more often had atrial fibrillation (20.7% vs 8.8%, *P* = .042), were more often previous smokers (53.3% vs 20.6%, *P* < .001), and had a higher body surface area (2.10 [SD 0.24] m^2^ vs 2.0 [SD 0.23] m^2^, *P* < .001) (Graphical Abstract). This group also had a significantly higher baseline LVEF and creatinine clearance, though both groups remained within normal ranges for cardiac and renal function (**Table 1**). The differences in baseline characteristics did not translate into differences in EuroSCORE II and Charlston Comorbidity Index (CCI) between the two groups, reflecting similar operative risk and burden of comorbidities (**Table 1**). Overall, there were missing data for the following variables: LVEF (10.6%), NYHA (2.5%), creatinine clearance (1.3%), body mass index (0.6%), and body surface area (0.6%). No patient was lost to follow-up. However, three patients migrated during the study period after a mean follow-up of 6.2 years (SD 1.8 years). After propensity score matching, the differences in baseline characteristics were balanced, with smoking status being the only parameter with significant difference between the groups, primarily driven by previous smoking status being more prevalent in the David population (56% vs 14%, *P* < .001).

**Table 1.** Baseline Characteristics for the Crude and Propensity Score-Matched Populations

	**Crude population**			Propensity score-matched population			
	David *N* = 92	Yacoub *N* = 68	*P*-value	David *N* = 50	Yacoub *N* = 50	*P*-value	Stand. diff.
Male, no (%)	75 (81.5)	49 (72.1)	.16	38 (76.0)	41 (82.0)	.46	0.15
Age (years), mean (SD)	49.7 (14.1)	51.5 (14.3)	.42	49.8 (14.0)	50.4 (14.9)	.84	−0.04
Body mass index (kg/m^2^), mean (SD)	25.5 (4.1)^41^	25.5 (4.1)	.11	25.1 (3.65)	25.7 (4.05)	.41	−0.17
Body surface area (m^2^), mean (SD)	2.1 (0.24) [1]	2.0 (0.23)	**<.001**	2.04 (0.22)	2.05 (0.21)	.67	−0.08
EuroSCOREII,^a^ median (IQR)	3.3 (2.5-4.1)	3.5 (2.7-4.9)	.12	3.4 (1.8-4.9)	3.4 (1.3-5.4)	.99	−0.13
Charleston comorbidity index, mean (SD)	0.24 (0.82)	0.37 (0.69)	.30	0.22 (0.93)	0.42 (0.73)	.24	−0.24
Left ventricular ejection fraction, no (%)	55 (8) [10]	54 (7) [3]	**.047**	54 (8) [1]	53 (8) [1]	.10	0.15
NYHA classification I-II/III-IV, no (%)	70/18 [4]	60/8	.15	41/9	45/5	.25	0.34
Hypertension, no (%)	50 (54.3)	36 (52.9)	.86	24 (48.0)	25 (50.0)	.84	0.04
Atrial fibrillation, no (%)	19 (20.7)	6 (8.8)	**.042**	4 (8.0)	6 (12.0)	.51	0.13
Diabetes mellitus, no (%)	2 (2.2)	3 (4.4)	.42	1 (2.0)	2 (4.0)	.56	0.12
Dyslipidaemia, no (%)	17 (18.5)	19 (27.9)	.16	8 (16.0)	12 (24.0)	.32	0.20
Previous myocardial infarction, no (%)	2 (2.2)	0	.22	2 (4.0)	0	.15	0.29
Previous cardiac surgery, no (%)	6 (6.5)	7 (10.3)	.39	3 (6.0)	5 (10.0)	.46	0.15
Peripheral vascular disease, no (%)	1 (1.1)	0	.39	0	0	N/A	N/A
Smoking status previous/active, no (%)	49/11	14/16	**<.001**	28/5	7/11	**<.001**	0.98
Creatinine clearance (mL/min), mean (SD)	124 (34.4) [1]	103 (34.9) [1]	**<.001**	117 (30.7)	108 (38.0) [1]	.21	0.26
Chronic obstructive pulmonary disease, no (%)	3 (3.3)	7 (10.3)	.07	1 (2.0)	5 (10.0)	.09	0.34
Connective tissue disease, no (%)	17 (18.5)	10 (14.7)	.59	10 (20.0)	9 (18.0)	.97	0.05

Missing values are reported in [] if any. Standardized mean differences are reported for comparisons between the two groups.

*P*-values <.05 are written in bold indicating significant differences.

a European System for Cardiac Operative Risk Evaluation (EuroSCORE) II is a score ranging from 0 to 100. The score indicates the percentual risk of death within 30 days after the procedure.

Abbreviations: NYHA, New-York Heart Association; Stand. Diff., standardized mean difference.

Overall, there were no difference in surgical indication for VSARR, valve morphology, and concomitant procedures between the two groups (**Table 2**). However, patients with David procedure were more often treated with larger tube graft sizes (≥30 mm) (David vs Yacoub; 55.4% vs 2.9%, *P* < .001), while patients with the Yacoub procedure more often had additional plications of the aortic valve (David vs Yacoub; 24 [26.1%] vs 47 [69.1%]; *P* < .001). Patients undergoing the Yacoub procedure also had shorter aortic cross-clamp time (David vs Yacoub; 156 [SD 46.7] min vs 125 [SD 25.1] min; *P* = .002), while there was no difference between the groups in extracorporeal circulation time (*P* = .26) or creatine kinase-MB max (*P* = .50). These significant differences between the two groups persisted in the PSM groups (**Table 2**).

**Table 2.** Operative Characteristics for the Crude and Propensity Score-Matched Populations

	Crude population			Propensity score-matched population		
	David *N* = 92	Yacoub *N* = 68	*P*-value	David *N* = 50	Yacoub *N* = 50	*P*-value
Aetiology, no (%)			.12			.76
Aortic aneurysm	19 (20.7)	6 (8.8)		7 (14.0)	5 (10.0)	
Aortic regurgitation	8 (8.7)	6 (8.8)		6 (12.0)	5 (10.0)	
Aneurism and regurgitation	65 (70.7)	56 (82.4)		37 (74.0)	40 (80.0)	
Tube graft size, no (%)	[7]		**<.001**	[3]		**<.001**
24 mm	0	9 (13.2)		0	2 (4.0)	
26 mm	8 (9.4)	26 (38.2)		5 (10.0)	21 (42.0)	
28 mm	25 (29.4)	31 (45.6)		16 (32.0)	26 (52.0)	
30 mm	39 (42.4)	2 (2.9)		21 (42.0)	1 (2.0)	
32 mm	13 (15.3)	0		5 (10.0)	0	
Supplemental procedures, no (%)						
Modified Yacoub w. annuloplasty	–	57 (83.8)	N/A	–	43 (86.0)	N/A
Plication	24 (26.1)	47 (69.1)	**<.001**	14 (28.0)	36 (72.0)	**<.001**
Aortic valve morphology, no (%)						
Bicuspid	17 (18.5)	16 (23.5)	.44	12 (24.0)	11 (22.0)	.81
Concomitant procedures, no (%)						
Antiarrhythmic procedures	5 (5.4)	1 (1.5)	.19	0	1 (2.0)	.32
Coronary artery bypass grafting	0	1 (1.5)	.24	0	1 (2.0)	.32
Mitral valve surgery	0	0	N/A	0	0	N/A
Aortic arch surgery	1 (1.1)	1 (1.5)	.83	0	0	N/A
Tricuspid valve surgery	0	0	N/A	0	0	N/A
Pulmonary valve surgery	1 (1.1)	1 (1.5)	.83	0	1 (2.0)	.32
Aortic cross-clamp time (minutes), mean (SD)	156 (46.7) [19]	125 (25.1) [41]	**.002**	158 (46.8) [10]	125 (28.1) [29]	**.005**
Extracorporeal circulation time (minutes), mean (SD)	203 (74.3) [19]	182 (44.0) [49]	.26	196 (68.5) [10]	184 (49.1) [35]	.52
Creatine kinase-MB max, median (IQR)	36.7 (26-69) [19]	43.0 (30-59) [19]	.50	37.5 (25-61) [12]	43.1 (30-63) [12]	.59

*P*-values <.05 are written in bold indicating significant differences.

Missing values are reported in [] if any. Standardized mean differences are reported for comparisons between the 2 groups.

Categorical data as *n* (percentage).

When comparing all elective VSARR procedures with all elective composite root replacement (CRR) procedures performed within the same study period, patients undergoing CRR were generally older (VSARR vs CRR, 50.4 [SD 14.1] years vs 54.1 [SD 14.1] years, *P* < .001) with a higher EuroSCORE II (3.3 [IQR 2.5-4.4] vs 3.7 [IQR 2.8-6.6], *P* < .001) and had more often undergone previous cardiac surgery (13 [8.1%] vs 78 [16.1%], *P* = .012). However, patients who had undergone VSARR more often had an underlying syndromic condition (27 [16.9%] vs 25 [5.1%], *P* < .001).

### Crude population

During a total follow-up time of 1231 person-years, with a median follow-up of 8.2 (SD 3.6) years for patients with the David procedure and 7.1 (SD 2.4) years for patients with the Yacoub procedure, we identified 12 patients, who died within the follow-up period, including seven patients (7.6%) in the David group and five patients (7.4%) in the Yacoub group (*P* = .95). Cardiovascular death occurred in five patients (5.4%) in the David group and three patients (4.4%) in the Yacoub group (*P* = .57) (**Table S5**).

The 5-year risk of death from all-causes was 2.2% (95% CI 0.6%-8.5%) for the David population and 6.0% (95% CI 2.3%-15.2%) for Yacoub population, while the 10-year estimates were 9.3% (95% CI 3.7%-22.1%) and 8.8% (95% CI 3.6%-20.6%), respectively, with no difference between the groups (log-rank *P* = .54) (Graphical Abstract). Sensitivity analysis between patients with the David procedure and the modified Yacoub procedure revealed no difference between the two groups regarding all-cause death (David 7.6% vs modified Yacoub 3.5%; *P* = .31).

**Figure 1:**
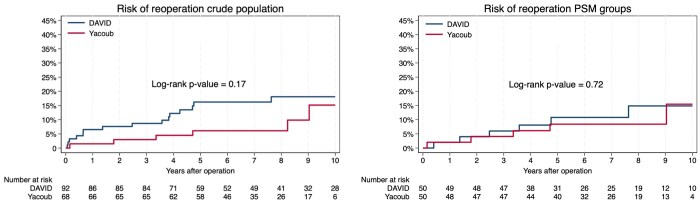
Risk of Reoperation in the Crude and the Propensity Score-Matched Population

**Figure 2:**
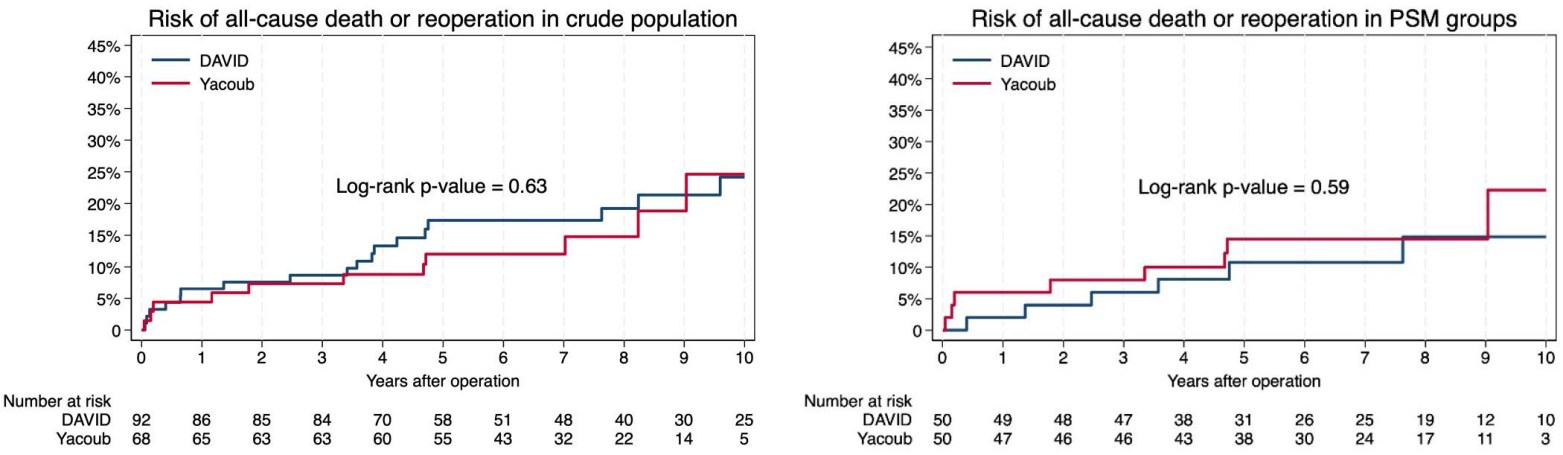
Risk of All-Cause Death or Reoperation in the Crude and the Propensity Score-Matched Population

The total reoperation rate was 17.4% (*n* = 16) and 8.8% (*n* = 6) within the David and Yacoub population, respectively, with no difference between the groups (*P* = .12) (**Table 3**). There were no aorta-related reoperations, while there was no difference in the incidence of valve-related reoperations (*P* = .12) or the timing of reoperation (*P* = .61) (**Table 3**). The 5-year risk estimates for reoperation were 16.2% (95% CI 9.9%-26.0%) and 6.1% (95% CI 2.3%-15.5%) for the David and Yacoub populations, respectively, while the 10-year risk estimates were 18.1% (95% CI 11.2%-28.5%) vs 15.2% (95% CI 6.2%-34.6%), with no difference between the groups (log-rank *P* = .17) (**Figure 1**). After combining death from all-causes and reoperation, there were still no difference in 5-year estimates (David vs Yacoub; 17.4% [95% CI: 10.8%-27.2%] vs 12.0% [95% CI: 6.2%-22.6%]) and 10-year estimates (David vs Yacoub; 24.2% [95% CI: 15.4%-36.7%) vs 24.6% (95% CI: 12.8%-44.2%)] (log-rank *P* = .63) (**Figure 2**). Sensitivity analysis between patients with the David procedure and the modified Yacoub procedure also revealed no difference between the two groups regarding reoperation (David 17.4% vs modified Yacoub 8.8%; *P* = .14).

**Table 3.** Data on Stroke and Reoperation in the Crude and Propensity Score-Matched Populations with Stratification According to Aortic Valve-Sparing Root Replacement Procedure

	**Crude population**			Propensity score-matched population		
	David *N* = 92	Yacoub *N* = 68	*P*-value	David *N* = 50	Yacoub *N* = 50	*P*-value
Total stroke, no. (%)	5 (5.4)	2 (2.9)	.76	2 (4.0)	1 (2.0)	.56
Stroke event timing, no. (%)			.30			.60
Periprocedural stroke^a^	1 (1.1)	2 (2.9)		1 (2.0)	1 (2.0)	
Early stroke^b^	1 (1.1)	0		1 (2.0)	0	
Late stroke^c^	3 (3.3)	0		0	0	
Stroke severity, no. (%)			.51			.60
Mild (NIHSS 0-5)	1 (1.1)	0		1 (2.0)	0	
Moderate (NIHSS 6-14)	2 (2.2)	2 (2.9)		1 (2.0)	1 (2.0)	
Severe (NIHSS ≥15)	2 (2.2)	0		0	0	
Total reoperation, no. (%)	16 (17.4)	6 (8.8)	.12	7 (14.0)	5 (12.0)	.54
Valve-related reoperation	16 (17.4)	6 (8.8)	.12	7 (14.0)	5 (10.0)	.54
Aorta-related reoperation	0	0	N/A	0	0	N/A
Reoperation timing, no. (%)			.61			.79
Periprocedural reoperation^d^	1 (1.1)	0		0	0	
Early reoperation^e^	5 (5.4)	1 (1.5)		1 (2.0)	1 (2.0)	
Late reoperation^f^	10 (10.9)	5 (7.4)		6 (12.0)	4 (8.0)	
Reoperation procedures, no. (%)			.43			.42
Isolated mechanical AVR	1 (6.3)	1 (16.7)		1 (14.3)	0	
Isolated biological AVR	2 (12.5)	0		2 (28.6)	0	
AVR, mechanical mitral valve and tricuspid annuloplasty	0	1 (14.3)		0	1 (16.7.0)	
Composite root replacement w. MVR	8 (50.0)	3 (42.9)		2 (28.6)	3 (50.0)	
Composite root replacement w. BVR	1 (6.3)	1 (14.3)		1 (14.3)	1 (16.7)	
Freestyle bioprosthesis	2 (12.5)	0		0	0	
TAVI	2 (12.5)	0		1 (14.3)	0	
Aortic tube graft	0	0		0	0	

*P*-values <.05 are written in bold indicating significant differences.

Missing values are reported in [] if any.

a Periprocedural stroke is defined as a stroke event occurring ≤30 days after the index procedure.

b Early stroke is defined as a stroke event occurring >30 days and ≤1 year after the index procedure.

c Late stroke is defined as a stroke event occurring >1 year after the index procedure.

d Periprocedural reoperation is defined as a reoperation event occurring ≤30 days after the index procedure.

e Early reoperation is defined as a reoperation event occurring >30 days and ≤1 year after the index procedure.

f Late reoperation is defined as a reoperation event occurring >1 year after the index procedure.

Abbreviations: AVR, aortic valve replacement; BVR, biological valve replacement; MVR, mechanical valve replacement; NIHSS, National Institute of Health Stroke Scale; TAVI, transcatheter aortic valve implantation.

The total incidence of stroke during follow-up was 5.4% and 2.9% within the David and Yacoub population, respectively, with no difference between the groups (*P* = .76) (**Table 3**). There were no differences between the groups regarding timing of stroke (*P* = .30) and stroke severity according to the National Institute of Health Stroke Scale (NIHSS) (*P* = .51) (**Table 3**).

### Elective VSARR vs elective CRR

When comparing patients treated with elective VSARR to patients treated with elective CRR (*n* = 486) from our database with end of follow-up the June 30, 2024,^10^ patients with VSARR had an overall lower all-cause death rate (VSARR vs CRR; 6.3% vs 12.6%, *P* = .027) and stroke rate (4.38% vs 9.67%, *P* = .036), while the reoperation rate was higher among VSARR (13.8% vs 4.3%; *P* < .001). However, there was no significant difference in risk of reoperation when accounting for competing risk of death (log-rank *P* = .17).

### Propensity score-matched population

A total of 50 patients from each VSARR group were matched 1:1 after PSM (**Table 1**). In the PSM population, we identified three patients, who died following VSARR, with no difference between the groups (**Table S5**). There was also no difference between the groups regarding death from all-causes, with similar 5- and 10-year risk estimates (David vs Yacoub; 0% [95% CI N/A] vs 6.9% [95% CI 2.2%-20.3%]; log-rank *P* = .09).

The total incidence of reoperation in the PSM population was 14.0% (*n* = 7) vs 10.0% (*n* = 5) within the David and Yacoub population, respectively, with no difference between the groups regarding valve-related reoperations (*P* = .54) and timing of reoperation (*P* = .79) (**Table 3**). The 5-year risk estimates for reoperation within the PSM groups were 10.8% (95% CI 4.6%-24.2%) for the David population and 8.4% (95% CI 3.2%-20.9%) for the Yacoub population, while 10-year risk estimates of reoperation were 14.9% (95% CI 6.7%-31.3%) vs 15.5% (95% CI 5.6%-38.6%) (log-rank *P* = .72) (**Figure 1**). After combining death from all-causes and reoperation in a composite end-point, there were still no difference between the groups, with 5-year risk estimates of 10.8% (95% CI: 4.6%-24.2%) vs 14.5% (95% CI: 7.2%-28.1%) for the David and Yacoub populations, respectively, and 10-year risk estimates of 14.9% (95% CI: 6.7%-31.3%) vs 22.3% (95% CI: 10.0%-45.3%) (log-rank *P* = .59) (**Figure 2**).

The total incidence of stroke in the PSM populations was 4.0% (*n* = 2) and 2.0 (*n* = 1) for the David and Yacoub procedures, respectively, with no difference between the groups (*P* = .56). There was also no difference between the groups regarding timing of stroke (*P* = .60) and stroke severity (*P* = .60) (**Table 3**).

#### Echocardiographic follow-up

 Echocardiographic follow-up was completed for 141 patients (88.1%) at baseline, while 138 patients (86.3%), 100 patients (62.5%), and 45 patients (28.1%) had echocardiographic follow-up data at discharge, after 3-5 years, and beyond 5 years, respectively (**Table 4**). The David population had significantly lower LVEF at echocardiographic follow-up 3-5 years postoperatively (David 55% [IQR: 50%-55%] vs Yacoub 55% [IQR: 55%-60%], *P* = .046), although median values in both the groups were within normal range. There were no differences in the number of patients with recurrent AR and AS and in the mean gradient of patients of patients with valve-related complications at all timepoints for echocardiographic follow-up (**Table 4**).

**Table 4.** Echocardiographic Follow-up of Patients in the Crude Population

Follow-up time	DAVID	Yacoub	P-value
Baseline	*N* = 83	*N* = 58	
LVEF, %—mean (SD)	55 (50-55) [9]	55 (50-55) [4]	.52
AR grade—no. (%)	[9]	[4]	.26
None/trace/mild	29 (34.9)	16 (27.6)	
Mild-moderate	4 (4.8)	4 (6.9)	
Moderate	9 (10.8)	12 (20.7)	
Moderate-severe	9 (10.8)	10 (17.2)	
Severe	32 (38.6)	16 (27.6)	
AS grade—no. (%)			**<.001**
None	82 (98.8)	57 (98.3)	
Mild	0	1 (1.7)	
Mild-moderate	0	0	
Moderate	0	0	
Moderate-severe	0	0	
Severe	1 (1.2)	0	
Mean gradient, mmHg^a^	– [1]	18	–
Discharge	*N* = 82	*N* = 56	
LVEF, %—median (IQR)	55 (55-55)	55 (50-55)	.33
AR grade—no. (%)			.76
None/trace/mild	77 (93.9)	54 (96.4)	
Mild-moderate	3 (3.7)	1 (1.8)	
Moderate	1 (1.2)	0	
Moderate-severe	0	0	
Severe	1 (1.2)	1 (1.8)	
AS grade—no. (%)			.41
None	81 (98.8)	56 (100)	
Mild	1 (1.2)	0	
Mild-moderate	0	0	
Moderate	0	0	
Moderate-severe	0	0	
Severe	0	0	
Mean Gradient, mmHg^a^	22	–	.22
3-5 years	*N* = 58	*N* = 42	
LVEF, %—median (IQR)	55 (50-55)	55 (55-60)	**.046**
AR grade—no. (%)			.65
None/trace/mild	36 (76.6)	23 (79.3)	
Mild-moderate	7 (14.9)	3 (10.3)	
Moderate	1 (2.1)	2 (6.9)	
Moderate-severe	0	0	
Severe	3 (6.4)	1 (3.5)	
AS grade—no. (%)			.25
None	56 (96.6)	40 (95.2)	
Mild	1 (1.7)	1 (2.4)	
Mild-moderate	0	1 (2.4)	
Moderate	0	0	
Moderate-severe	0	0	
Severe	1 (1.7)	0	
Mean gradient, mmHg—mean (SD)^a^	47 (24)	35 (15)	.44
End of follow-up (>5 years)	*N* = 30	*N* = 15	
Years from surgery, mean (SD)	9.0 (2.5)	7.3 (1.5)	
LVEF, %—median (IQR)	55 (55-55)	55 (50-55)	.48
AR grade—no. (%)			.44
None/trace/mild	26 (86.7)	11 (73.3)	
Mild-moderate	2 (6.7)	1 (6.7)	
Moderate	2 (6.7)	2 (13.3)	
Moderate-severe	0	0	
Severe		1 (6.7)	
AS grade—no. (%)			.15
None	30 (100)	14 (93.3)	
Mild	0	1 (6.7)	
Mild-moderate	0	0	
Moderate	0	0	
Moderate-severe	0	0	
Severe	0	0	
Mean gradient, mmHg—mean (SD)^a^	–	– [1]	

Missing values are reported in [] if any. *P*-values <.05 are written in bold indicating significant differences.

a Mean gradients are only reported for patients with mild, moderate, moderate-severe or severe AS at the different follow-up points.

Abbreviations: AR, aortic regurgitation; AS, aortic stenosis: LVEF, left ventricular ejection fraction.

### Cox regression model

In a univariate model, baseline characteristic associated with increased mortality included higher age, EuroSCORE II, and CCI along with the presence of hypertension, diabetes mellitus, dyslipidaemia, and chronic obstructive pulmonary disease. Higher creatinine clearance was the only parameter associated with a significant reduction in risk of all-cause death (**Table S6**). After adjustment, diabetes mellitus (HR 16.9 [95% CI 1.56-184]) and chronic obstructive pulmonary disease (HR 157 [95% CI 8.61-2897]) were the only independent factors associated with increased mortality, with no baseline characteristics being associated with significantly reduced mortality (**Table S6**). Year of surgery did not associate with mortality.

## DISCUSSION

In this nationwide, multicentre, population-based cohort study, VSARR with the David and Yacoub techniques yielded comparable long-term outcomes, despite few persistent differences in baseline characteristics even after PSM. All-cause death, reoperation, and stroke rates did not differ, and echocardiographic follow-up showed preserved valve function in both the groups. Additionally, *post hoc* analyses comparing elective patients treated with CRR to the crude study population showed significantly lower rates of all-cause death and stroke among patients treated with VSARR, without any difference in the risk of reoperation after accounting for the competing risk of death. These findings extend evidence from high-volume centres to a low-volume national setting, underscoring that durable results can be achieved when procedures are performed by experienced teams.^23^^,^^24^

With a median follow-up of 8.2 years, we reported an overall all-cause death rate of 7.5% and a reintervention rate of 14.4%, with mid- and long-term risk estimates consistent with results from international single- and multicentre cohorts^25–27^ and a recent meta-analysis.^7^ Importantly, the absence of excess reoperation or valve dysfunction supports both techniques as reliable in real-world practice, even when performed outside high-volume programs. Thus, our study offers insights into real-world, multicentre data, which have undergone a meticulous validation and adjudication process to secure the highest quality of data and robustness of our findings.^9^

The David and Yacoub procedures represent two distinct, yet closely related, approaches in the surgical management of aortic root disease. VSARR procedures have continuously undergone modifications and expanded indications to include patients with bicuspid aortic valves, complex congenital heart disease, and those with previous cardiac surgery.^28^ However, each case requires careful consideration of many aspects, including the surgical indication, comorbidities, and anatomy of the aortic valve and root, all of which inform and guide risk discussion with the patient. The choice between the David and Yacoub procedure is often highly dependent on the centre and the surgeon’s preference and experience with each procedure, which is also reflected in the varying distribution of VSARR procedures at the three centres in our study. However, contemporary literature seems to reflect a growing emphasis on the David procedure, especially in the setting of younger patients, patients with Marfan syndrome, and patients with bicuspid aortic valves.^29–31^ This observed favouritism might reflect the improved annular support provided by the subannular suture line in the David procedure. Studies of preoperative predictors of recurrent AR and reoperation have shown that larger annular dimensions, Marfan syndrome, aortic dissection, and cusp repair/plication increase the risk of reintervention or valve failure.^3^^,^^32–34^ Subgroup analyses further demonstrated that larger annuli and Marfan syndrome were associated with higher risk of postoperative AR after a Yacoub procedure, indicating that these patients may be better suited for the David procedure.^32^^,^^35^ Therefore, it is important that we demonstrate comparable results across all outcomes between the two procedures, and that connective tissue disease and bicuspid aortic valve were not associated with all-cause death.

The issue regarding increased risk of postoperative annular dilatation have led to the development of the modified Yacoub procedure with an external ring.^4^ In our study, the modified procedure was performed in 83.8% of cases with remodelling and was selectively employed in the presence of abnormal annular dimensions identified during intraoperative assessment. Since the modification was invented, studies have been published with varying results; some have reported comparable results,^35^ while others observed an increased risk of reintervention compared to the David procedure after six years follow-up.^36^ Our study thus adds knowledge to this specific topic, demonstrating comparable results between the two procedures without increased risk of reoperation following remodelling with or without an external ring. However, one study has recently shed light on the risk of reoperation after 20-year follow-up after the modified Yacoub procedure, highlighting cases with Marfan syndrome, in which root dilatation reoccurred despite annuloplasty with an external ring.^30^ This finding raises the question whether or not the aortic annulus is the only anatomic structure at risk of recurrent dilatation. Further studies with larger multi-centre cohorts and long-term follow-up need to be conducted to fully address this issue.

Contrary to the Yacoub procedure, the David procedure resuspend the native aortic valve within a synthetic graft, which replaces the aortic root completely.^1^^,^^3^ The replacement of the entire aortic root along with the subannular suture line has raised some concerns about changes haemodynamic root and annular physiology, potentially leading to altered valve dynamics and blood distribution with an associated risk of earlier valve deterioration. The modified David V procedure was then proposed by De Paulis, using a Dacron conduit with incorporated sinuses of Valsalva, which short-term yielded satisfactory outcomes.^37^^,^^38^ In Denmark, the Valsalva graft is widely used when performing the David procedure, which adds to the current knowledge on long-term outcomes after the David V procedure. In this context, it is also important that the echocardiographic follow-up revealed only one case of >mild postoperative AS, which was caused by a subvalvular obstruction rather than valve deterioration.

While our findings align with the evolving consensus favouring valve repair over replacement, most studies on VSARR have been conducted in high-volume centres.^8^^,^^25^^,^^26^^,^^39^^,^^40^ In Denmark, the total annual procedures ranged from three to 23 procedures and 42 to 81 procedures for VSARR and CRR, respectively, with few senior consultants performing the procedures at four different centres, resulting in an institutional and individual caseload below the proposed definition of high-volume.^9^^,^^40^ This study uniquely evaluates its feasibility in a lower-volume, national setting, reinforcing its potential as a preferred strategy for appropriately selected patients. Although VSARR is a highly specialized procedure, CRR also requires highly experienced surgeons. The recommendations on intervention for severe AR are not entirely clear-cut, so increasing availability of valve-sparing procedures may influence the timing of intervention.^8^ We believe that preoperative imaging including electrocardiogram-gated cardiac CT and transoesophageal echocardiography further supports patient selection and is among the contributing factors. Future research may explore institutional learning curves to better understand these dynamics to help optimize training, patient selection, and long-term results. Clearly defining patient selection criteria is essential, particularly in the early phases of VSARR adoption, as precise selection may help establish VSARR as the preferred treatment option.

### Limitations

This study has several important limitations that must be considered. First, as a retrospective observational study, it is subject to inherent biases, including missing data and selection bias. Despite propensity matching, residual confounding cannot be excluded. The decision between the David and Yacoub procedures may still have been influenced by unmeasured patient characteristics, clinical factors, frailty, or surgeon-specific preferences not captured in the available data. PSM was employed to mitigate indication bias, but it cannot fully substitute for the benefits of randomization in a controlled trial. The relatively small sample size limited subgroup analyses (eg, Marfan, bicuspid valves) and precluded robust assessment of surgeon- or centre-specific effects.

Second, the Kaplan-Meier method used to estimate the incidence of stroke and reoperation does not account for competing risks such as all-cause death. As a result, these event rates may be overestimated, while lower number of patients in the long-term follow-up may have contributed to higher 10-year risk estimates. To address this, we also analysed a composite end-point that included each of these events combined with all-cause death to provide a more comprehensive assessment.

Third, an important limitation of this study is the lack of standardized echocardiographic measurements, which introduces variability in the assessment of valve function.

Despite these limitations, our dataset captured all Danish tertiary centres, and underwent a thorough validation process, creating a unique dataset with high data completeness, validity, and robust long-term outcome assessment.^9^

## CONCLUSION

In this nationwide population-based study, both VSARR procedures were associated with satisfactory rates of all-cause death, stroke, and reoperation. There was also no difference in the timing and severity of stroke, the timing and reason for reoperation, and in postoperative echocardiographic measurements. Our findings support both VSARR techniques as viable long-term surgical strategies in carefully selected patients, underscoring the importance of management within highly specialized cardiac and aortic teams with extensive experience in the chosen procedure.

## Supplementary Material

ivag107_Supplementary_Data

## Data Availability

The data supporting this article were obtained from the WDHR and the DHR, which are not publicly available due to Danish data protection regulations. Data may be available from the authors upon reasonable request and with approval from the relevant data protection authorities.

## ABBREVIATIONS

AR: Aortic regurgitation
AS: Aortic stenosis
DHR: Danish Heart Registry
ICD: International Classification of Diseases
LVEF: Left ventricular ejection fraction
NIHSS: National Institute of Health Stroke Scale
PSM: Propensity score-matched
VSARR: Valve-sparing aortic root replacement
WDHR: Western Danish Heart Registry
