# Supplementary material for: Valve-Sparing Aortic Root Replacement: Comparison of Long-Term Outcomes Between the David and Yacoub Procedure in Denmark
Source: Interdiscip Cardiovasc Thorac Surg. 2026 Apr 10;41(5):ivag107. doi: 10.1093/icvts/ivag107 (PMC13143430; doi:10.1093/icvts/ivag107)
Supplement: ivag107_Supplementary_Data [file ivag107_supplementary_data.docx]

# **Supplemental material**

**Long-term outcomes after David or Yacoub valve-sparing aortic root replacement: A Danish multi-center cohort study**

Table of content:

**Tables**

- Page 2: Supplemental Table S1 – Definitions and codes of endpoints
- Page 3: Supplemental Table S2 - Coding algorithms and weights for the Charlson Comorbidity Index 1987 model
- Page 5: Supplemental Table S3 – Propensity score-matching analyses
- Page 6: Supplemental Table S4 - Baseline characteristics for the crude elective VSARR and CRR populations
- Page 7: Supplemental Table S5 – Causes of death by surgical procedure within the crude and propensity score-matched population
- Page 8: Supplemental Table S6 – Association between covariables and all-cause death for the crude population

**Supplemental Table S1** – ICD10 and SKS codes of endpoints and definitions of endpoints

| **Data** | **Outcome** | **Code** |
| --- | --- | --- |
| **SKS-codes** | Aortic root replacement with either composite root replacement or aortic valve-sparing root replacement | KFCA50, KFCA60, KFCA70, KFCA96, KFCA96, KFMD, KFMC00, KFMC10, KFMC20 |
| **ICD10-codes** | Stroke | DI60, DI61, DI62, DI63, DI64 |
|  | Reoperation | KFMD, KFCA50, KFCA60, KFCA70, KFCA96^*^ |
| **Definitions** | Stroke | All patients with ICD10-codes for stroke were evaluated by review of medical records. Each case was either confirmed/rejected and graded according to the National Institutes of Health Stroke Scale (NIHSS). |
|  | Dyslipidemia | Patients with active statin treatment prior to surgery and/or documented dyslipidemia in medical records |
|  | Hypertension | Patients with active treatment with antihypertensive agents prior to surgery and/or documented hypertension in medical records |
|  | Diabetes mellitus | Patients with active treatment with antidiabetic agents prior to surgery and/or documented diabetes mellitus in medical records |
|  | Atrial fibrillation | Patients with active treatment with antiarrhythmic agents prior to surgery and/or documented atrial fibrillation in medical records from electrocardiographic measurements |
|  | Chronic obstructive pulmonary disease | Patients with active treatment prior to surgery and/or documented chronic obstructive pulmonary disease in medical records |
|  | Connective tissue diseases | Patients with confirmed connective tissue disease based on multidisciplinary evaluation including physical examinations, gene testing and/or biopsy |
|  | End of follow up | All patients are followed until 24^th^ of November, 2025 |

^*^Patients registered with any of these ICD10-codes after the date of primary surgery

| **Supplemental Table S2 -** Coding algorithms and weights for the Charlson comorbidities index 1987 model^1^ | | |
| --- | --- | --- |
| **Comorbidities** | **ICD-10^2^** | **Weight** |
| Myocardial infarction | I21.x, I22.x, I25.2 | 1 |
| Congestive heart failure | I09.9, I11.0, I13.0, I13.2, I25.5, I42.0, I42.5–I42.9, I43.x, I50.x, P29.0 | 1 |
| Peripheral vascular disease | I70.x, I71.x, I73.1, I73.8, I73.9, I77.1, I79.0, I79.2, K55.1, K55.8, K55.9, Z95.8, Z95.9 | 1 |
| Cerebrovascular disease | G45.x, G46.x, H34.0, I60.x–I69.x | 1 |
| Dementia | F00.x–F03.x, F05.1, G30.x, G31.1 | 1 |
| Chronic pulmonary disease | I27.8, I27.9, J40.x–J47.x, J60.x–J67.x, J68.4, J70.1, J70.3 | 1 |
| Rheumatic disease | M05.x, M06.x, M31.5, M32.x–M34.x, M35.1, M35.3, M36.0 | 1 |
| Peptic ulcer disease | K25.x–K28.x | 1 |
| Mild liver disease | B18.x, K70.0–K70.3, K70.9, K71.3–K71.5, K71.7, K73.x, K74.x, K76.0, K76.2–K76.4, K76.8, K76.9, Z94.4 | 1 |
| Diabetes without chronic complication | E10.0, E10.1, E10.6, E10.8, E10.9, E11.0, E11.1, E11.6, E11.8, E11.9, E12.0, E12.1, E12.6, E12.8, E12.9, E13.0, E13.1, E13.6, E13.8, E13.9, E14.0, E14.1, E14.6, E14.8, E14.9 | 1 |
| Diabetes with chronic complication | E10.2–E10.5, E10.7, E11.2–E11.5, E11.7, E12.2–E12.5, E12.7, E13.2– E13.5, E13.7, E14.2–E14.5, E14.7 | 2 |
| Hemiplegia or paraplegia | G04.1, G11.4, G80.1, G80.2, G81.x, G82.x, G83.0–G83.4, G83.9 | 2 |
| Renal disease | I12.0, I13.1, N03.2–N03.7, N05.2– N05.7, N18.x, N19.x, N25.0, Z49.0–Z49.2, Z94.0, Z99.2 | 2 |
| Any malignancy, including lymphoma and leukemia, except malignant neoplasm of skin | C00.x–C26.x, C30.x–C34.x, C37.x– C41.x, C43.x, C45.x–C58.x, C60.x–C76.x, C81.x–C85.x, C88.x, C90.x–C97.x | 2 |
| Moderate or severe liver disease | I85.0, I85.9, I86.4, I98.2, K70.4, K71.1, K72.1, K72.9, K76.5, K76.6, K76.7 | 3 |
| Metastatic solid tumor | C77.x–C80.x | 6 |
| AIDS/HIV | B20.x–B22.x, B24.x | 6 |
| ICD codes were identified within 5-year period prior to the intervention. The same comorbidities with different severity were mutually exclusive: diabetes with chronic complications and diabetes without chronic complications; mild liver disease and moderate or severe liver disease; and any malignancy and metastatic solid tumor. | | |

^1^Charlson, 1987: Charlson ME, Pompei P, Ales KL, MacKenzie CR. A new method of classifying prognostic comorbidi-ty in longitudinal studies: development and validation. J Chronic Dis. 1987;40(5):373-383. doi:10.1016/0021-9681(87)90171-8

^2^Quan, 2005: Quan H, Sundararajan V, Halfon P, et al. Coding Algorithms for Defining Comorbidities in ICD-9-CM and ICD-10 Administrative Data. Medical Care. 2005;43(11):1130-1139. doi:10.1097/01.mlr.0000182534.19832.83

| **Supplemental Table S3** - Propensity score matching analyses | |
| --- | --- |
| **Outcome** | EuroSCORE 2 |
| **Treatment** | DAVID / Yacoub |
| **Covariates (n=5)** | Age |
|  | Body mass-index |
|  | Body surface area |
|  | Atrial fibrillation |
|  | Left ventricular dysfunction |
| **Number of observations** | 160 |
| **Caliper** | 0·2 of the logit^1,2^ |
| **Matched** | 1:1 |
| **PScore generated population, n** | 50 / 50  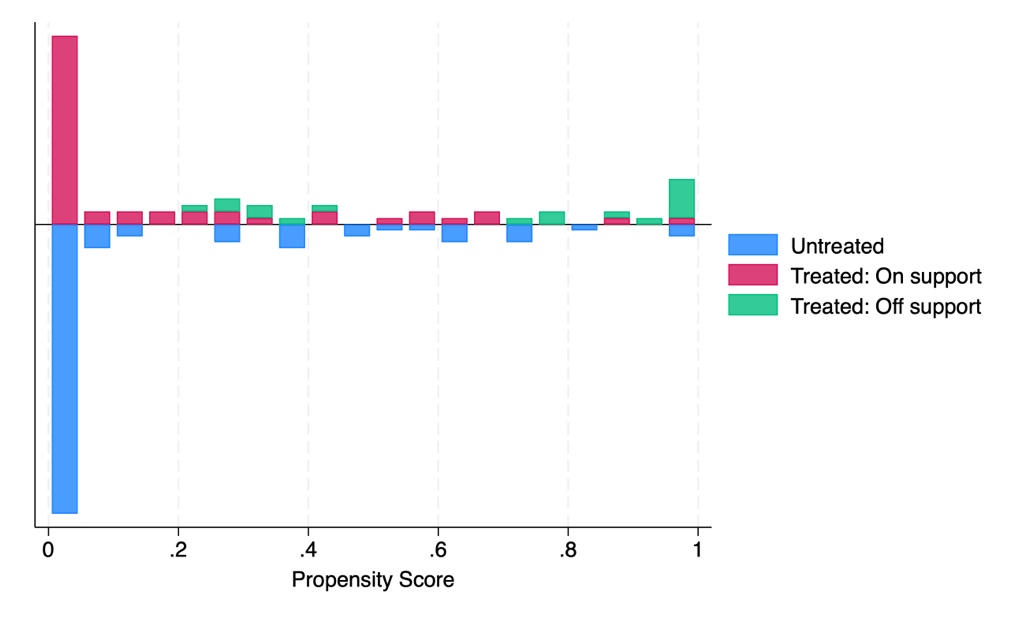 |

1) Austin PC. Optimal caliper widths for propensity-score matching when estimating differences in means and differences in proportions in observational studies. Pharm Stat. 2011 Mar-Apr;10(2):150-61. doi: 10.1002/pst.433. PMID: 20925139; PMCID: PMC3120982.

2) Kuss O, Blettner M, Börgermann J. Propensity Score: an Alternative Method of Analyzing Treatment Effects. Dtsch Arztebl Int. 2016 Sep 5;113(35-36):597-603. doi: 10.3238/arztebl.2016.0597. PMID: 27658473; PMCID: PMC5963493.

**Supplemental Table S4 -** Baseline characteristics for the crude elective VSARR and CRR populations

|  | **Crude population** | | |
| --- | --- | --- | --- |
|  | **VSARR  N = 160** | **CRR**  **N = 486** | **p-value** |
| Male, no (%) | 124 (77.5) | 399 (82.1) | 0.19 |
| Age (years), mean (SD) | 50.4 (14.1) | 54.1 (14.1) | **0.004** |
| Body mass index(kg/m^2^), mean (SD) | 26.1 (4.1) [1] | 26.7 (4.4) | 0.08 |
| Body surface area (m^2^), mean (SD) | 2.1 (0.24) [1] | 2.1 (0.24) [11] | 0.59 |
| EuroSCOREII^a^, median (IQR) | 3.3 (2.5-4.4) | 3.7 (2.8-6.6) | **<0.001** |
| Charleston Comorbidity Index, mean (SD) | 0.29 (0.77) | 0.44 (1.04) | 0.09 |
| Left ventricular ejection fraction, no (%) | 54 (8) [13] | 54 (10) [40] | 0.44 |
| NYHA classification I-II / III-IV, no (%) | 134/26 [4] | 351/112 | 0.05 |
| Hypertension, no (%) | 86 (53.8) | 269 (55.4) | 0.72 |
| Atrial fibrillation, no (%) | 25 (15.6) | 78 (16.1) | 0.90 |
| Diabetes mellitus, no (%) | 32 (6.6) | 5 (3.1) | 0.10 |
| Dyslipidemia, no (%) | 36 (22.5) | 125 (25.7) | 0.41 |
| Previous myocardial infarction, no (%) | 2 (1.3) | 11 (2.3) | 0.43 |
| Previous cardiac surgery, no (%) | 13 (8.1) | 78 (16.1) | **0.012** |
| Peripheral vascular disease, no (%) | 1 (0.6) | 9 (1.9) | 0.28 |
| Smoking status previous / active, no (%) | 63/27 | 174/100 | 0.53 |
| Creatinine clearance (ml/min), mean (SD) | 115.0 (36.0) [2] | 107 (36.0) [19] | **0.018** |
| Chronic Obstructive pulmonary disease, no (%) | 10 (6.3) | 33 (6.8) | 0.81 |
| Connective tissue disease, no (%) | 27 (16.9) | 25 (5.1) | **<0.001** |

Missing values are reported in [] if any. Standardized mean differences are reported for comparisons between the two groups.

**^a^**European System for Cardiac Operative Risk Evaluation (EuroSCORE) II is a score ranging from 0 to 100. The score indicates the percentual risk of death within 30 days after the procedure.

Abbreviations: CRR, Composite Root Replacement; NYHA, New-York Heart Association; VSARR, Valve-Sparing Aortic Root Replacement

P-values <0.05 are written in **bold** indicating significant differences.

| **Supplemental Table S5** – Causes of death by surgical procedure within the crude population and PSM group | | | | | | | |
| --- | --- | --- | --- | --- | --- | --- | --- |
| **Cause of Death** | **Crude population** | | |  | **Propensity score-matched cohort** | | |
|  | **DAVID**  N = 92 | **Yacoub** N = 68 | **p-value** |  | **DAVID**  N = 50 | **Yacoub** N = 50 | **p-value** |
| **Cardiovascular death, no. (%)** | 5 (5.4) | 3 (4.4) | 0.77 |  | 0 | 2 (4.0) | 0.15 |
| Sudden cardiac death | 2 (2.2) | 3 (4.4) | 0.42 |  | 0 | 2 (4.0) | 0.15 |
| **Cancer, no. (%)** | 1 (1.1) | 2 (2.9) | 0.39 |  | 0 | 1 (2.0) | 0.32 |
| **Pulmonary failure, no. (%)** | 0 | 0 | N/A |  | 0 | 0 | NA |
| **Cerebral disease, no. (%)** | 1 (1.1) | 0 | 0.39 |  | 0 | 0 | N/A |
| **Others, no. (%)** | 0 | 0 | N/A |  | 0 | 0 | N/A |
| **Total deaths, no. (%)** | 7 (7.6) | 5 (7.4) | 0.95 |  | 0 | 3 (6.0) | 0.08 |

P-values <0.05 are written in **bold** indicating significant differences.

**Supplemental Table S6** – Association between covariables and all-cause death for the crude population

| **Crude population** | **Univariable analyses** | | **Multivariable analysis** | |
| --- | --- | --- | --- | --- |
|  | **Hazard Ratio (95% CI)** | **p-value** | **Hazard Ratio (95% CI)** | **p-value** |
| David procedure, yes | 1.83 (0.52-6.55) | 0.35 |  |  |
| Male, yes | 0.50 (0.16-0.1.58) | 0.24 | 0.10 (0.00-2.23) | 0.15 |
| Age, years | 1.15 (1.06-1.24) | **0.001** | 1.10 (0.97-1.25) | 0.14 |
| Body mass index, kg/m^2^ | 1.13 (0.99-1.31) | 0.08 | 0.81 (0.58-1.12) | 0.20 |
| Body surface area, m^2^ | 1.57 (0.14-17.5) | 0.72 | 128 (0.22-76097) | 0.14 |
| EuroSCOREII^a^, % | 1.24 (1.01-1.51) | **0.037** | 0.71 (0.44-1.13) | 0.15 |
| Charlson Comorbidity index, % | 1.91 (1.31-2.78( | **0.001** | 2.59 (0.80-8.36) | 0.11 |
| Left ventricular ejection fraction, % | 0.97 (0.91-1.05) | 0.49 |  |  |
| NYHA classification III or IV, yes | 3.02 (0.85-10.7) | 0.09 |  |  |
| Hypertension, yes | 4.30 (0.94-19.7) | **0.06** | 7.58 (0.65-88.0) | 0.11 |
| Atrial fibrillation, yes | 2.58 (0.77-8.64) | 0.12 |  |  |
| Diabetes Mellitus, yes | 13.0 (3.42-49.5) | **<0.001** | 16.9 (1.56-184) | **0.020** |
| Dyslipidemia, yes | 3.56 (1.14-11.1) | **0.029** | 7.23 (0.77-67.5) | 0.08 |
| *Previous myocardial infarction, yes | - | N/A |  |  |
| *Previous cardiac surgery, yes | - | N/A |  |  |
| *Peripheral vascular disease, yes | - | N/A |  |  |
| Smoking, yes | 1.72 (0.46-6.39) | 0.42 |  |  |
| Creatinine clearance, ml/min | 0.97 (0.95-0.99) | **0.004** | 0.99 (0.94-1.03) | 0.58 |
| Chronic Obstructive pulmonary disease, yes | 7.06 (1.86-26.8) | **0.004** | 157 (8.61-2897) | **0.001** |
| Connective tissue disease, yes | 0.24 (0.03-1.87) | 0.17 | 3.23 (0.12-90.8) | 0.49 |
| *Bicuspid valve, yes | - | N/A |  |  |
| Year of surgery | 1.00 (1.00-1.00) | 0.61 |  |  |

*Hazard ratio could not be estimated due to few / no cases

Abbreviations: NYHA, New-York Heart Association

P-values <0.05 are written in **bold** indicating significant differences.

No interaction between all-cause death and connective tissue disease, previous endocarditis, or aortic valve-sparing root replacement.
